# A Novel Systematic Oxidative Stress Score Predicts the Prognosis of Patients with Operable Breast Cancer

**DOI:** 10.1155/2021/9441896

**Published:** 2021-10-07

**Authors:** Kaiming Zhang, Liqin Ping, Tian Du, Yan Wang, Ya Sun, Gehao Liang, Xi Wang, Xiaoming Xie, Weidong Wei, Xiangsheng Xiao, Jun Tang

**Affiliations:** ^1^Department of Breast Oncology, Sun Yat-sen University Cancer Center, State Key Laboratory of Oncology in South China, Collaborative Innovation Center for Cancer Medicine, Guangzhou, Guangdong, China; ^2^Department of Medical Oncology, Sun Yat-sen University Cancer Center, State Key Laboratory of Oncology in South China, Collaborative Innovation Center for Cancer Medicine, Guangzhou, Guangdong, China

## Abstract

**Background:**

Breast cancer was associated with imbalance between oxidation and antioxidation. Local oxidative stress in tumors is closely related to the occurrence and development of breast cancer. However, the relationship between systematic oxidative stress and breast cancer remains unclear. This study is aimed at exploring the prognostic value of systematic oxidative stress in patients with operable breast cancer.

**Methods:**

A total of 1583 operable female breast cancer patients were randomly assigned into the training set and validation set. The relationship between systematic oxidative stress biomarkers and prognosis were analyzed in the training and validation sets.

**Results:**

The systematic oxidative stress score (SOS) was established based on five systematic oxidative stress biomarkers including serum creatinine (CRE), serum albumin (ALB), total bilirubin (TBIL), lactate dehydrogenase (LDH), and blood urea nitrogen (BUN). SOS was an independent prognostic factor for operable breast cancer patients. A nomogram based on SOS and clinical characteristics could accurately predict the prognosis of operable breast cancer patients, and the area under the curve (AUC) of the nomogram was 0.823 in the training set and 0.872 in the validation set, which was much higher than the traditional prognostic indicators.

**Conclusions:**

SOS is an independent prognostic indicator for operable breast cancer patients. A prediction model based on SOS could accurately predict the outcome of operable breast cancer patients.

## 1. Background

Breast cancer is a malignancy with the highest incidence and the highest mortality rate among female population [1]. The heterogeneity of breast cancer is strong, and the prognosis of patients with the same American Joint Committee on Cancer (AJCC) stage and immunohistochemical subtype is greatly different [2, 3], suggesting that there are still subtle factors affecting the outcome of patients despite known risk factors such as AJCC stage and immunohistochemical subtype.

Reactive oxygen species (ROS) are important mediators for the body's defense and killing cancer cells. However, excessive ROS could induce DNA damage and genomic instability, leading to the loss of cell integrity, function, and viability [4]. Breast cancer cells have a higher level of ROS, and DNA damage induced by ROS is closely related to the occurrence and development of breast cancer [5–7].

At present, the important role of local ROS in tumor tissue has been reported by many studies [8–10], but the relationship between systematic oxidative stress and prognosis of breast cancer patients is still unclear. In a systematic oxidative stress mouse model, total bilirubin (TBIL), lactate dehydrogenase (LDH), creatinine (CRE), and blood urea nitrogen (BUN) were significantly increased [11]. At the same time, in critically ill polytrauma patients, there were significant statistical differences in TBIL, serum albumin (ALB), LDH, and C-reactive protein (CRP) of patients with antioxidant treatment compared with those without [12]. It was suggested that these biochemical indicators may be biomarkers of systematic oxidative stress of the whole body.

This study is aimed at exploring the relationship between systematic oxidative stress and prognosis of breast cancer patients based on biochemical oxidative indicators. Furthermore, a novel systematic oxidative stress score (SOS) was established based on biochemical indicators of systematic oxidative stress. Finally, a prognostic nomogram was created by combining SOS with other clinical variables for predicting the prognosis of breast cancer patients.

## 2. Methods

### 2.1. Patients and Study Design

This study included female breast cancer patients who received breast conserving surgery or modified radical mastectomy at Sun Yat-sen University Cancer Center (SYSUCC) from August 2012 to December 2015. Inclusion criteria included the following: (1) diagnosed as invasive breast cancer by histopathology, (2) received breast conserving surgery or modified radical mastectomy at SYSUCC, (3) no distant metastasis before surgery, (4) complete preoperative biochemical examination and clinical information, and (5) normal liver and kidney function. Exclusion criteria included the following: (1) received preoperative chemotherapy or other antitumor therapy before surgery, (2) complicated with other uncured malignant tumors, and (3) renal or liver dysfunction. A total of 1583 female breast cancer patients were enrolled in the study and randomly assigned to the training set (*n* = 1187) or validation set (*n* = 396). Prognostic models were established in the training set and the accuracy of the prediction model was subsequently verified in the validation set. The study was approved by the SYSUCC Ethics Committee (identifier: 81372133), and all patients signed an informed consent form. The study complied with the Helsinki Declaration and the Ethics Committee.

### 2.2. Data Collection and Treatment

We collected the information about age, gender, history of disease, immunohistochemical (IHC) subtype of breast cancer, number of primary lesions, tumor size and T stage, lymph node metastasis and N stage, AJCC stage and vessel carcinoma embolus (VCE) of breast cancer patients from SYSUCC hospital information system. CRE, TBIL, direct bilirubin (DBIL), LDH, uric acid (UA), BUN, ALB, and CRP were obtained 3 days before operation. The biochemical indicators were measured by automatic biochemical analyzer (Hitachi Ltd. 7600 Serial, Tokyo, Japan). According to the receiver operating characteristic (ROC) curve analysis, the best cut-off values of these biochemical indicators were as follows: CRE 59.3 *μ*mol/L, DBIL 2.2 *μ*mol/L, TBIL 13.8 *μ*mol/L, LDH 205.7 U/L, UA 255.2 *μ*mol/L, BUN 6.29 mmol/L, ALB 43 g/L, and CRP 3.37 mg/L. And the status of these biochemical indicators was defined as high if it was greater than the cut-off, and low if not. Age was defined as the age of the patient at the time of surgery. The sex is the biological sex. Complicated with other uncured tumors refers to the occurrence of other malignancies within 5 years (excluding curable carcinoma in situ). Pathological diagnosis of enrolled patients was reviewed by an experienced pathologist at SYSUCC, and patients were pathologically analyzed based on IHC and/or fluorescence in situ hybridization (FISH) detection of estrogen receptor (ER), progesterone receptor (PR), human epidermal growth factor receptor-2 (HER-2), and Ki-67 status. The size of the tumor was the longest diameter of the tumor tissue reported by pathology. The number of lymph node metastasis was determined by postoperative histopathological examination. Distant metastases preoperatively were identified by radiographic examination. The AJCC staging of patients is based on the Eighth Edition of the AJCC Staging Systems. Most patients received standard postoperative adjuvant chemotherapy, anti-HER2 therapy, postoperative adjuvant radiotherapy, or postoperative adjuvant endocrine therapy. They were followed up and received physical check every three months after surgery, every six months after two years, and every year after five years. The overall survival (OS) of patients who died was defined as the time from surgical treatment to death, and the OS of patients who are still alive was defined as the time from surgery to the last follow-up. The last follow-up of patients enrolled in this study was in December 2020.

### 2.3. Statistical Analysis

R software version 4.0.2 (R Statistical Computing Foundation, Vienna, Austria) and SPSS 24.0 (IBM, Armonk, NY, USA) were used to perform the analysis of this research. Chi-squared test was used to analyze the differences in proportions of clinical variables. The independent prognostic indicators of OS were identified through univariate Cox regression analysis and multivariate Cox regression analysis. Then, five optimal biochemical indicators, such as CRE, ALB, TBIL, LDH, and BUN, were identified for calculating systematic oxidative stress score (SOS), which was based on the lowest Akaike information criterion (AIC) value [13]. The SOS of each patient was calculated by the status of biochemical indicators, which below the cut-off value was defined as 0, and above the cut-off value was defined as 1. The corresponding regression coefficient of each biochemical indicator was identified based on multivariate Cox regression analysis. The formula of SOS was as follows: SOS = sum (corresponding regression coefficient × status of biochemical indicator). Patients were separated into low-SOS and high-SOS groups based on the median value of the SOS. ‘rms' package of R software was used to construct a nomogram combining SOS with other clinical variables. The predictive performance of the nomogram was analyzed through calibration plots. The time-dependent ROC curve was used to evaluate the predictive accuracy of nomogram. *P* value < 0.05 in two-tailed test was considered statistically significant.

## 3. Results

### 3.1. Clinical Characteristics of Patients

A total of 1583 female breast cancer patients were enrolled in this study, of whom 1187 (75%) patients were randomly assigned to the training set, and 396 (25%) patients were assigned to the validation set. The clinical characteristics as well as the preoperative biochemical oxidative stress markers of breast cancer patients in the training set and validation set were shown in Table 1. There were no statistical differences in clinical characteristics and systematic oxidative stress indicators between the two sets.

### 3.2. Systematic Oxidative Stress Score (SOS) Was Established Based on Systematic Oxidative Stress Indicators

**Figure 1:**
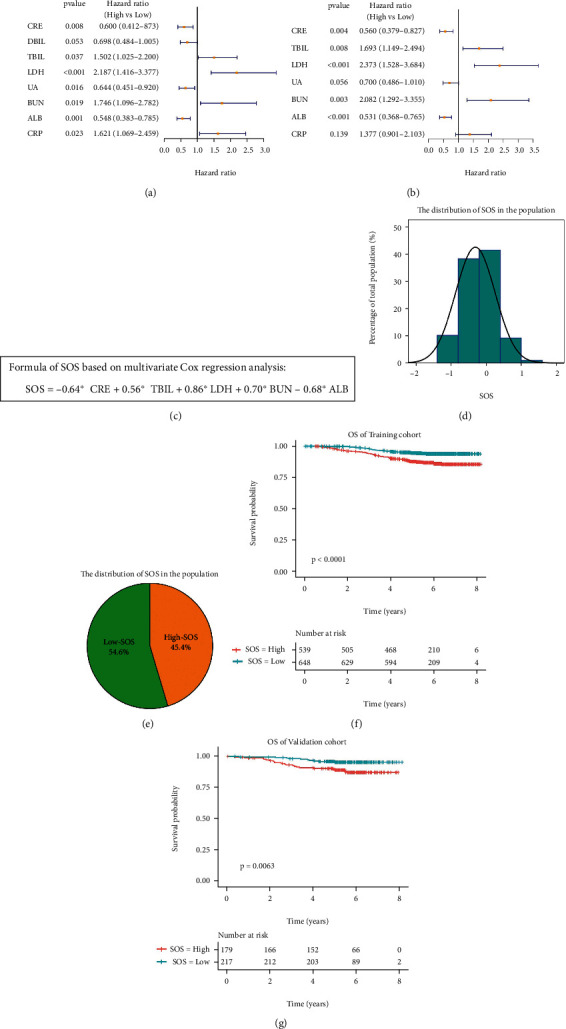
SOS is related with OS of breast cancer patients. (a, b) Systematic oxidative stress indicators were included in the univariate Cox regression analysis and multivariate Cox regression analysis in the training set. (c) The specific calculation formula of SOS. (d, e) The distribution of SOS in breast cancer patients. (f) Kaplan-Meier curves showed that the OS of high-SOS patients was longer than that of low-SOS patients in the training set. (g) Kaplan-Meier curves showed that the OS of high-SOS patients was longer than that of low-SOS patients in the validation set.

To explore the prognostic value of systematic oxidative stress indicators, the systematic oxidative stress indicators were transformed into dichotomous variables according to the cut-off determined by ROC. In univariate Cox regression analysis, CRE, TBIL, LDH, UA, BUN, ALB, and CRP were correlated with the OS of breast cancer patients, while there is no statistical relationship between DBIL and OS (Figure 1(a)). In multivariate Cox regression analysis, elevated CRE and ALB were associated with better OS, while elevated TBIL, LDH, and BUN predicted worse prognosis (Figure 1(b)). To simplify the calculation, the status of systematic oxidative stress indicator was defined as 1 if it was greater than the cut-off, and 0 if not. Then, the systematic oxidative stress score (SOS) was established based on multivariate Cox regression analysis and the lowest value of AIC (Table 2). Finally, the formula of SOS was as follows: SOS = −0.64 × CRE + 0.56 × TBIL + 0.86 × LDH + 0.7 × BUN − 0.68 × ALB (Figure 1(c)). The distribution of SOS in breast cancer patients was shown in Figure 1(d). The value of SOS was between -1.32 and 1.56, and the median SOS was -0.12. Based on the median SOS, breast cancer patients were divided into the high-SOS group (45.4%) and low-SOS group (56.6%) (Figure 1(e)). Patients with higher SOS had worse prognosis in the training set (Figure 1(f)), and the same result was observed in the validation set (Figure 1(g)).

### 3.3. The Relationship between Systematic Oxidative Stress Score (SOS) and Clinical Characteristics

**Figure 2:**
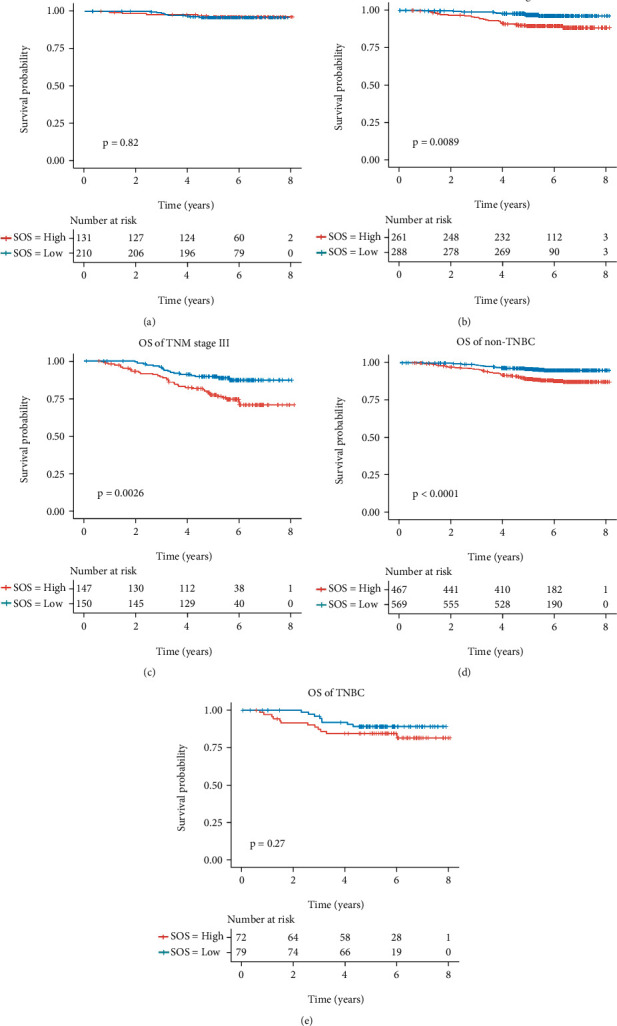
Subgroup-based survival analysis for patients with breast cancer. (a) Kaplan-Meier analysis for the OS of patients with AJCC staging I breast cancer. (b) Kaplan-Meier analysis for the OS of patients with AJCC staging II breast cancer. (c) Kaplan-Meier analysis for the OS of patients with AJCC staging III breast cancer. (d) Kaplan-Meier analysis for the OS of patients with nontriple-negative breast cancer. (e) Kaplan-Meier analysis for the OS of patients with triple-negative breast cancer.

The relationship between SOS and clinical characteristics was shown in Table 3, and we could find that elder or advanced T stage patients tended to have higher SOS. Then, subgroup analysis was performed in breast cancer patients with different clinical characteristics. In patients with stage II and III breast cancer, there was a statistical difference in OS between the low-SOS and high-SOS groups (Figures 2(b) and 2(c)). However, no statistical difference was observed in stage I breast cancer patients (Figure 2(a)). In terms of immunohistochemical subtypes, the higher SOS in patients with non-triple-negative breast cancer predicted a worse prognosis (Figure 2(d)). But there was no statistical difference in prognosis between the high-SOS and low-SOS patients with triple-negative breast cancer (Figure 2(e)).

### 3.4. SOS Was an Independent Prognostic Indicator of OS for Breast Cancer Patients

Univariate and multivariate Cox regression analyses were performed to determine the independent prognostic value of SOS. The result of univariate Cox regression analysis showed that age, SOS, histological grade, VCE, T stage, N stage, and IHC subtype were prognostic indexes for OS. Subsequently, these indexes were included in the multivariate Cox regression analysis, and the results showed that age, SOS, T stage, N stage, and IHC subtype were independent prognostic indicators (Table 4). In general, SOS was an independent prognostic indicator of OS for breast cancer patients.

### 3.5. Construction and Verification of a Nomogram

**Figure 3:**
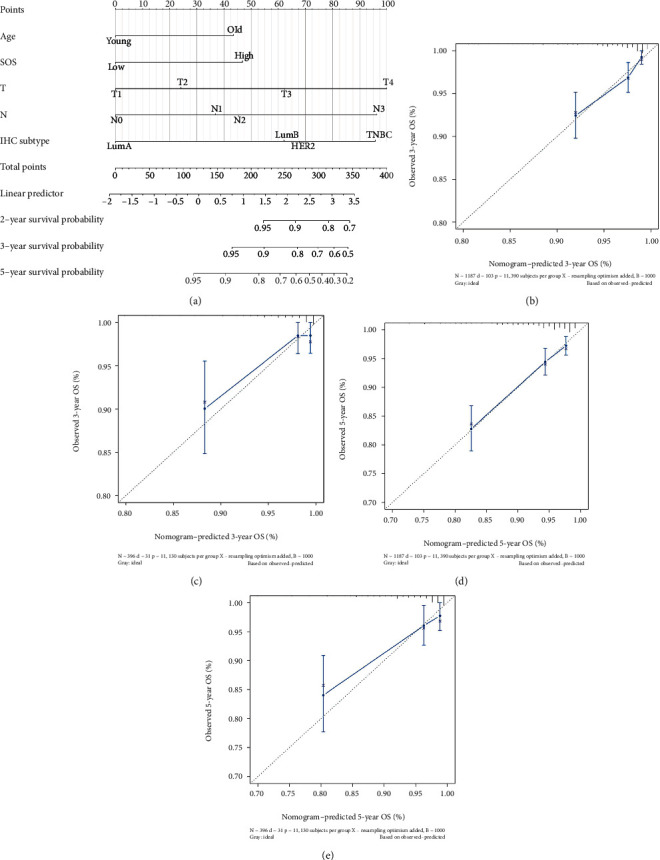
Nomogram could predict the OS of breast cancer patients. (a) Nomogram for predicting the OS of patients with breast cancer. (b) Calibration plot of the nomogram for 3-year overall survival in the training cohort. (c) Calibration plot of the nomogram for 3-year overall survival in the validation cohort. (d) Calibration plot of the nomogram for 5-year overall survival in the training cohort. (e) Calibration plot of the nomogram for 5-year overall survival in the validation cohort.

**Figure 4:**
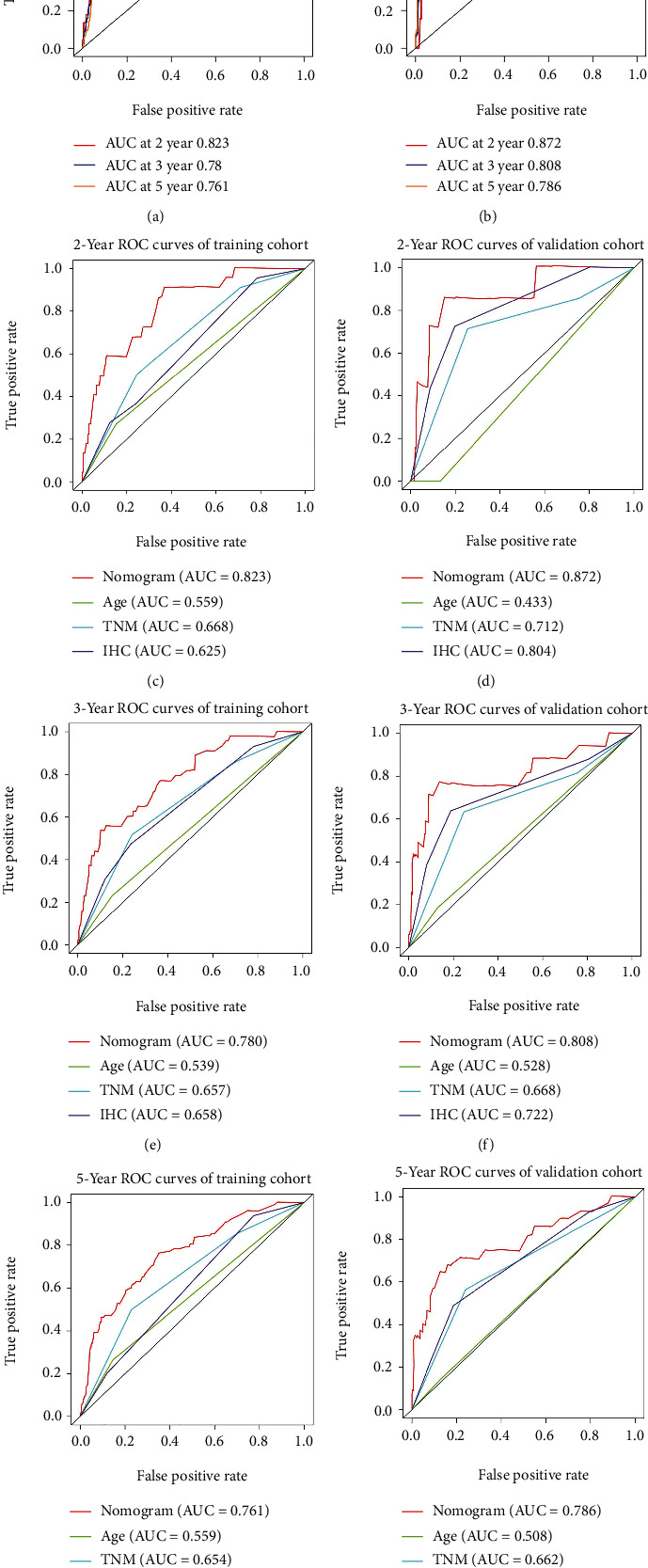
The predictive accuracy of our nomogram is much better than that of previous prognostic indicators. (a). Time-dependent ROC curves were used to determine the prognostic value of the nomogram in the training set. (b) Time-dependent ROC curves were used to determine the prognostic value of the nomogram in the validation set. (c–h) Area under the ROC curves was used to compared the prognostic value of the nomogram and previous prognostic indicators for predicting 2-year overall survival, 3-year overall survival, or 5-year overall survival in the training set and validation set.

All independent prognostic indexes identified through multivariate Cox regression analysis were included to build a nomogram (Figure 3(a)). The consistency of this nomogram was analyzed by calibration plots, showing the strong consistency between prediction and observation in predicting OS (Figures 3(b)–3(e)). And the predictive accuracy was estimated by time-dependent ROC curve analysis, showing that the area under the curve (AUC) of the nomogram in predicting 2-year, 3-year, and 5-year survival rates were 0.823, 0.780, and 0.761, respectively, in the training set and were 0.872, 0.808 and 0.786, respectively, in the validation set (Figures 4(a) and 4(b)). In order to compare the predictive capacity between this nomogram and traditional prognostic indicators, such as age, AJCC staging, and IHC subtype, the ROC curve analysis was conducted. And the results showed that the AUC of the nomogram was much higher than that of these traditional prognostic indicators (Figures 4(c)–4(h)), suggesting that the nomogram owned a higher accuracy in predicting OS than these traditional prognostic indicators.

## 4. Discussion

This study is the first to explore the significance of systematic oxidative stress status for the prognosis of breast cancer and the first to establish a prognostic model including SOS. In this study, biochemical indicators of systematic oxidative stress were analyzed to explore the relationship between systematic oxidative stress and prognosis in breast cancer patients. According to univariate and multivariate Cox regression analyses, elevated CRE and ALB were associated with better OS, while elevated TBIL, LDH, and BUN predicted poorer prognosis. In order to combine these factors, SOS was established based on these five indicators. SOS was an independent prognostic indicator for breast cancer patients, and higher SOS was related with poorer survival. Then, a nomogram based on SOS and clinical characteristics was built, which could provide higher accuracy in predicting OS than traditional prognostic indicators.

The imbalance between oxidation and antioxidation is related to the occurrence and development of breast cancer. The high-risk factors of breast cancer, such as age increasing, obesity, alcohol consumption, smoking, estrogen, BRCA gene mutation, and ionizing radiation, are all related to oxides and oxidative stress [14]. As an important tumor suppressor gene, BRCA1 gene is involved in the upregulation of gene expression of protective antioxidant response and antioxidant response transcription factors. Its products can downregulate the level of ROS in cells and protect cells from DNA oxidative damage [5]. On the contrary, estrogen can lead to DNA damage and the occurrence of breast cancer by inducing the generation of ROS [7].

Studies on the prevention of breast cancer have shown that dietary intake of high antioxidant foods was associated with a lower risk of breast cancer [15]. The increased intake of vegetable-fruit-soybean diet in postmenopausal women was associated with a dose-dependent decrease in breast cancer risk [16]. Oxidative stress promotes the formation of breast cancer, but the relationship between the level of systematic oxidative stress and the prognosis of breast cancer patients remains unclear. Some studies reported that antioxidants can reduce the effectiveness of treatment and even contribute to the progression of breast cancer. Tamoxifen (TAM) induced apoptosis in MCF-7 cells by inducing the increase of ROS and the release of proapoptotic factors in mitochondria. However, vitamin C, an antioxidant, can protect cancer cells from TAM-induced oxidation, thereby inhibiting MCF-7 cell death [17]. Meanwhile, vitamin E, another antioxidant, could significantly reduce the production of ROS and the expression of p53 to promote the proliferation of MCF-7 cells [18]. It was suggested that the relationship between oxidative stress and breast cancer was complex. Before tumor formation, excessive oxidants can lead to DNA damage and increase the incidence of cancer. However, once the tumor was formed, the reduced oxidation level caused by antioxidants may reduce the ability of killing cancer cells, leading to the progression of cancer and the decrease of therapeutic efficacy.

Bilirubin is the end product of heme metabolism and is considered an anticancer factor due to its antioxidant function, but the relationship between bilirubin and prognosis of cancers is adverse. Evidence showed increased bilirubin indicated poorer prognosis in advanced non-small-lung cancer, cholangiocarcinoma, and rectal cancer patients [19–21]. In patients with metastatic breast cancer, higher bilirubin levels are associated with decreased survival [22]. In this study, elevated total bilirubin was associated with worse prognosis for breast cancer patients. In muscle, creatine is formed into CRE and released into the blood through an irreversible nonenzymatic dehydration reaction. Endogenous CRE is a product of muscle metabolism in the human body. BUN is the main end product of protein metabolism in human body. Both CRE and BUN are excreted by the kidneys, but systematic oxidative stress reduces the ability of the kidneys to excrete urea and creatinine, leading to increases of CRE and BUN in blood, which decrease after antioxidant treatment [23]. Therefore, CRE and BUN could reflect the status of systematic oxidative stress. It has been proved that antioxidant compounds could activate the antioxidant transcription factor Nrf2 and reduce CRE level [24, 25]. On the other hand, the synthesis of BUN is the main way to reduce ammonia. If the synthesis of BUN is blocked, it will lead to the increase of ammonia, which could promote the production of ROS and oxidative stress [26]. In this study, increased CRE and decreased BUN were associated with longer OS in breast cancer patients. ALB is an important protein produced by the liver and can reflect the nutritional status and inflammatory response status of the human body [27]. In addition, ALB has antioxidant function and enzymatic activity [28]. Higher ALB levels are associated with longer OS in patients with a number of cancers, including breast cancer [29]. It is consistent with the results of our study. Lactate dehydrogenase A (LDHA) provides energy for tumor metabolism by promoting glycolysis to transform pyruvate into lactic acid. LDHA produces *α*-hydroxybutyrate and triggers hypermethylation of histone H3K79, which activates the antioxidant reaction [30]. Decrease of LDHA translation or inhibition of LDHA function can reduce ATP production, increase ROS production, and induce significant oxidative stress and cell death [31]. In our study, decreased LDH was associated with longer OS in breast cancer patients.

To our knowledge, all reported biochemical markers associated with oxidative stress were included in this study, including CRE, TBIL, LDH, UA, BUN, ALB, and CRP. After univariate and multivariate analyses, we identified five independent systematic oxidative stress indicators (TBIL, BUN, CRE, ALB, and BUN) to calculate SOS, which is an independent prognostic factor for breast cancer patients. This study reported the relationship between systematic oxidative stress indexes and breast cancer prognosis for the first time and established the prediction model, of which the AUC could reach 0.872.

Oxidative stress is involved in the formation and development of tumors, but there are few reports on its effect on prognosis. SOS can provide prognostic information for patients from systematic oxidative stress status. This study could help clinicians identify patients with poor outcomes, allowing more aggressive treatment regimens for high-risk patients, and increasing the frequency of postoperative follow-up. In addition, the results of this study are beneficial to the subsequent studies on the relationship between oxidative stress and tumor prognosis and provide reference for the development of therapeutic targets for oxidative stress. However, our study was a single-center retrospective study, and the causal relationship between the included indicators and oxidative stress as well as the mechanism is still unclear, which still needs to be confirmed by multicenter prospective studies and more basic research in the future.

## 5. Conclusion

SOS can predict the prognosis of patients with breast cancer based on the status of oxidative stress, and the SOS-based nomogram has a good accuracy in predicting the prognosis of patients with operable breast cancer. In addition, SOS provides a new idea for the establishment of prognosis model of breast cancer.

## Figures and Tables

**Table 1:** Clinical characteristics in the training set and validation set.

Variables	Total (*n* = 1583)		Training set (*n* = 1187)		Validation set (*n* = 396)		*P* value
	No.	%	No.	%	No.	%	
Age							0.252
≤60	1347	85.1	1003	84.5	344	86.9	
>60	236	14.9	184	15.5	52	13.1	
Multifocality							0.927
Yes	39	2.5	29	2.4	10	2.5	
No	1544	97.5	1158	97.6	386	97.5	
Histological grade							0.180
I	108	6.8	89	7.5	19	4.8	
II	903	57.0	671	56.5	232	58.6	
III	572	36.2	427	36.0	145	36.6	
VCE							0.307
Yes	558	35.2	410	34.5	148	37.4	
No	1025	64.8	777	65.5	248	62.6	
T stage							0.371
T1	717	45.3	549	46.2	168	42.4	
T2	760	48.0	555	46.8	205	51.8	
T3	60	3.8	47	4.0	13	3.3	
T4	46	2.9	36	3.0	10	2.5	
N stage							0.248
N0	828	52.3	630	53.1	198	50.0	
N1	402	25.4	298	25.1	104	26.3	
N2	207	13.1	145	12.2	62	15.7	
N3	146	9.2	114	9.6	32	8.0	
TNM stage							0.263
Stage I	438	27.7	341	28.7	97	24.5	
Stage II	744	47.0	549	46.3	195	49.2	
Stage III	401	25.3	297	25.0	104	26.3	
IHC subtype							0.155
Luminal A	328	20.7	252	21.3	76	19.2	
Luminal B	883	55.8	645	54.3	238	60.1	
HER2+	184	11.6	139	11.7	45	11.4	
TNBC	188	11.9	151	12.7	37	9.3	
CRE (*μ*mol/L)							0.544
<59.30	963	60.8	717	60.4	246	62.1	
≥59.30	620	39.2	470	39.6	150	37.9	
DBIL (*μ*mol/L)							0.326
<2.20	393	24.8	302	25.4	91	23.0	
≥2.20	1190	75.2	885	74.6	305	77.0	
TBIL (*μ*mol/L)							0.262
<13.80	1270	80.2	960	80.9	310	21.7	
≥13.80	313	19.8	227	19.1	86	78.3	
LDH (U/L)							0.732
<205.70	1424	90.0	1066	89.8	358	90.4	
≥205.70	159	10.0	121	10.2	38	9.6	
UA (*μ*mol/L)							0.357
<255.20	408	25.8	299	25.2	109	27.5	
≥255.20	1175	74.2	888	74.8	287	72.5	
BUN (mmol/L)							0.466
<6.29	1424	90.0	1064	89.6	360	90.9	
≥6.29	159	10.0	123	10.4	36	9.1	
ALB (g/L)							0.966
<43.00	830	52.4	622	52.4	208	52.5	
≥43.00	753	47.6	565	47.6	188	47.5	
CRP (mg/L)							0.807
<3.78	1361	86.0	1022	86.1	339	85.6	
≥3.78	222	14.0	165	13.9	57	14.4	

**Table 2:** Multivariate Cox regression analysis of candidate indicators for SOS.

Factors	Coef	HR (95% CI)	*P* value
CRE	-0.64	0.525 (0.358-0.769)	0.000966
TBIL	0.56	1.756 (1.194-2.582)	0.004186
LDH	0.86	2.365 (1.527-3.663)	0.000114
BUN	0.7	2.017 (1.256-3.240)	0.003678
ALB	-0.68	0.506 (0.353-0.726)	0.000221

**Table 3:** Relationship between SOS and clinical characteristics in the training set.

Variables	Total (n = 1187)		SOS high (*n* = 539)		SOS low (*n* = 648)		*P* value
	No.	%	No.	%	No.	%	
Age							0.002
≤60	1003	84.5	436	80.9	567	87.5	
>60	184	15.5	103	19.1	81	12.5	
Multifocality							0.115
Yes	29	2.4	9	1.7	20	3.1	
No	1158	97.6	530	98.3	628	96.9	
Histological grade							0.611
I	89	7.5	36	6.7	53	8.2	
II	671	56.5	306	56.8	365	56.3	
III	427	36.0	197	36.5	230	35.5	
VCE							0.790
Yes	410	34.5	184	34.1	226	34.9	
No	777	65.5	355	65.9	422	65.1	
T stage							0.038
T1	549	46.2	225	41.7	324	50.0	
T2	555	46.8	275	51.0	280	43.2	
T3	47	4.0	23	4.3	24	3.7	
T4	36	3.0	16	3.0	20	3.1	
N stage							0.059
N0	630	53.1	264	49.1	366	56.5	
N1	298	25.1	142	26.3	156	24.0	
N2	145	12.2	74	13.7	71	11.0	
N3	114	9.6	59	10.9	55	8.5	
IHC subtype							0.466
Luminal A	252	21.1	104	19.3	148	22.8	
Luminal B	645	54.3	296	54.9	349	53.9	
HER2+	139	11.7	67	12.4	72	11.1	
TNBC	151	11.9	72	13.4	79	12.2	

**Table 4:** Results of the univariate and multivariate Cox regression analyses for OS among the clinical characteristics and SOS.

Variables	Univariate Cox analysis		Multivariate Cox analysis	
	HR (95% CI)	*P* value	HR (95% CI)	*P* value
Age		0.002		0.003
≤60	Reference		Reference -	
>60	1.435 (1.155-1.783)		1.943 (1.247-3.027)	
SOS		<0.001		<0.001
Low	Reference		Reference	
High	2.389 (1.589-3.592)		2.096 (1.385-3.170)	
Multifocality		0.786		
No	Reference			
Yes	1.177 (0.373-3.712)			
Histological grade		0.019		0.430
I	Reference		Reference	
II	1.704 (0.616-4.716)	0.305	1.411 (0.495-4.023)	0.520
III	2.719 (0.980-7.542)	0.055	1.749 (0.602-5.082)	0.304
VCE		<0.001		0.055
No	Reference		Reference	
Yes	2.295 (1.558-3.380)		1.518 (0.992-2.322)	
T stage		<0.001		<0.001
T1	Reference		Reference	
T2	2.076 (1.310-3.290)	0.002	1.409 (0.876-2.265)	0.157
T3	4.812 (2.328-9.945)	<0.001	2.558 (1.190-5.497)	0.016
T4	7.158 (3.548-14.442)	<0.001	4.528 (2.150-9.540)	<0.001
N stage		<0.001		<0.001
N0	Reference		Reference	
N1	1.680 (0.999-2.825)	0.050	1.567 (0.911-2.693)	0.104
N2	2.211 (1.217-4.017)	0.009	1.634 (0.856-3.121)	0.137
N3	5.911 (3.586-9.741)	<0.001	3.612 (2.056-6.346)	<0.001
IHC subtype		<0.001		0.032
Luminal A	0.185 (0.078-0.439)	<0.001	0.261 (0.107-0.642)	0.003
Luminal B	0.684 (0.414-1.131)	0.139	0.618 (0.362-1.056)	0.078
HER2+	0.683 (0.340-1.373)	0.284	0.625 (0.305-1.283)	0.201
TNBC	Reference		Reference	

## Data Availability

These data are available by individual application to the corresponding authors.
